# Ripening Study Based on Multi-Structural Inversion of Cherry Tomato qMRI

**DOI:** 10.3390/foods13244056

**Published:** 2024-12-16

**Authors:** Yanan Li, Jingfa Yao, Wenhui Yang, Zhao Wei, Peng Luan, Guifa Teng

**Affiliations:** 1 College of Information Science & Technology, Hebei Agricultural University, Baoding 071001, China; liyanan@hebau.edu.cn (Y.L.);; 2 Hebei Key Laboratory of Agricultural Big Data, Baoding 071001, China; 3 Hebei Software Engineering Department, Baoding 071030, China; 4 Hebei College Intelligent Interconnection Equipment and Multi-Modal Big Data Application Technology Research and Development Center, Baoding 071030, China; 5 Institute of Electrical Engineering, Chinese Academy of Sciences, Zhongguancun Beiertiao NO.6, Beijing 100190, China; 6 University of Chinese Academy of Sciences, Beijing 100049, China; 7 Instrumental Analysis Center, Shanghai Jiao Tong University, Shanghai 200240, China; 8 Hebei Digital Agriculture Industrial Technology Research Institute, Shijiazhuang 056400, China

**Keywords:** ripening, low-field MRI, T2 relaxation inversion, structural, moisture

## Abstract

This study introduces a non-destructive, quantitative method using low-field MRI to assess moisture mobility and content distribution in cherry tomatoes. This study developed an advanced 3D non-local mean denoising model to enhance tissue feature analysis and applied an optimized TransUNet model for structural segmentation, obtaining multi-echo data from six tissue types. The structural T2 relaxation inversion was refined by integrating an ACS-CIPSO algorithm. This approach addresses the challenge of low signal-to-noise ratios in multi-echo MRI images from low-field equipment by introducing an innovative solution that effectively reduces voxel noise while retaining structural relaxation variability. The study reveals that there are consistent patterns in the changes in moisture mobility and content across different structures of cherry tomatoes during their ripening process. Mono-exponential analysis reveals the patterns of changes in moisture mobility (T2) and content (A) across various structures. Furthermore, tri-exponential analysis elucidates the patterns of changes in bound water (T21), semi-bound water (T22), and free water (T23), along with their respective contents. These insights offer a novel perspective on the changes in moisture mobility throughout the ripening process of tomato fruit, thereby providing a research pathway for the precise assessment of moisture status and ripening expression in fruits.

## 1. Introduction

Cherry tomato (*Lycopersicon esculentum* Var. Cerasiforme Alef.) is a small tomato variety native to the Central American region; it is now widely cultivated and consumed worldwide due to its sweet and sour taste and richness in nutrients such as minerals and vitamins. The moisture status and ripeness of cherry tomatoes directly affect their taste, flavor, and nutritional value [1,2]. The correlation analysis of the moisture status and ripeness of tomatoes to accurately assess their ripeness is important for the identification and analysis of their quality traits [3,4].

In recent years, extensive research has been conducted on the detection of fruit ripening. Conventional ripeness assessment techniques for fruits primarily utilize optical, acoustic, thermal, and electrical properties, which exhibit certain limitations. Optical methods, such as visible light and spectral analysis, are highly subjective and susceptible to appearance and color [5,6,7], with extensive data processing required for hyperspectral analysis [8]. Acoustic methods suffer from low interpretability and are easily affected by external acoustic environments [9,10]. Thermal methods also have low interpretability and are sensitive to external temperature variations [11]. Electrical methods may cause damage to the fruit and result in unstable measurement outcomes [12,13].

Nuclear magnetic resonance technologies, encompassing nuclear magnetic resonance spectroscopy (NMR) and magnetic resonance imaging (MRI), have seen increased application in the non-destructive testing of fruits. The development of low-field NMR equipment with low cost, easy maintenance, and straightforward operation has been a driving force behind this advancement [14]. Nuclear magnetic resonance technology acquires information based on the relaxation behavior of hydrogen (H) protons within the sample. The state of H protons in fruit cells serves as an intrinsic parameter indicative of fruit texture. The T2 relaxation technique is the most expedient for assessing the phase state of fruits, where the extent of water interaction with other substances is determined by measuring T2 relaxation times [15]. Samples are categorized based on T2 relaxation times into different states, which include free water (the longest T2), semi-bound water, and bound water (the shortest T2), with the water content proportion in each state deduced from their relative ratios. [16]. Leveraging the strengths of relaxation analysis for examining moisture phase states and dynamics, the T2 relaxation technique has successfully elucidated the distribution and condition of water within various fruits during physiological alterations and processing, with examples including development [17], ripening [3,18,19,20], dehydration [14], and thermal processing [21]. NMR data express the overall relaxation data of the samples and prevent the obtaining of information about the spatial location inside the samples [22,23,24,25,26]. Magnetic resonance imaging (MRI), performed under a gradient magnetic field, has the ability to provide highly resolved spatial information on the magnetic environment. This imaging modality captures both water content and spatial details without characterizing the state of the water, enabling the visualization of tissue microstructure and the distribution of water content [27,28].

Quantitative magnetic resonance imaging (qMRI) utilizes T2-weighted imaging of multiple acquired echoes. Based on the principle of nuclear magnetic relaxation, the exponential decay curve obtained from these echoes is fit to yield the T2 relaxation time and the corresponding amplitude for each voxel. This inversion process provides spatial information related to T2 relaxation and its amplitudes, resulting in T2 mapping and amplitude maps [29]. Typically, a mono-exponential inversion of the T2 mapping corresponds to the water mobility of the sample voxel [30]. Due to the diverse water present in fruit tissues, mono-exponential T2 mapping fails to accurately depict tissue microstructure and composition. The multi-exponential T2 relaxation model distinguishes multiple moisture components with varying T2 relaxation times, thereby reflecting the intricate water distribution within tissues [4]. The triple-exponential inversion method for T2 mapping has been studied and has proven effective for analyzing water states in fruits [30,31,32,33].

NMR and MRI have been utilized in the ripening analysis of tomato-based fruits [3]. An MRI-weighted image analysis study demonstrated a strong correlation between the MRI characteristics of various cherry tomato cultivars and their ripeness [34]. Musse’s investigation of tomato structural NMR measurements through cut-sample experiments revealed water redistribution among cellular compartments during ripening, presenting good consistency in the water states in different structures of the tomato at different ripening stages [4]. The voxel-wise triple-exponential inversion method for T2 mapping has been studied [30,33] and proven effective for analyzing water states in fruits [18].

This research involved acquiring multi-echo MRI images from diverse cherry tomato varieties at various stages of ripeness. The study enhanced image processing and inversion algorithms to address the low signal-to-noise ratio typical of low-field MRI systems. Subsequent structured T2 multi-exponential inversion enabled the extraction of T2 relaxation data from each fruit structure, providing insights into the hydration levels of the fruits’ internal structures across different ripening stages.

## 2. Materials and Methods

**Figure 1:**
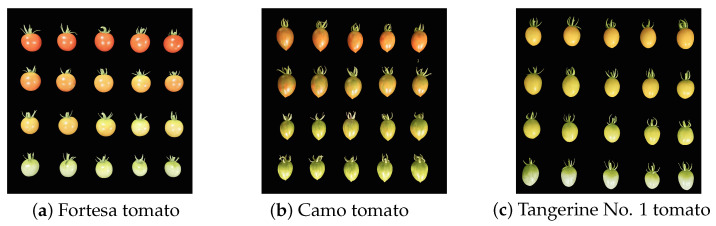
Cherry tomato sample display.

Samples: The experimental samples were picked in mid-July 2023 at the tomato breeding base of Hebei Agricultural University, Hebei Province, China. Three varieties of cherry tomatoes (Fortesa tomato, Camo tomato, and Tangerine No. 1 tomato) were collected for the experiment, with four ripening periods (from the bottom to the top: green ripening, color change, firm ripening, and full ripening), as shown in Figure 1. Five samples of each were subjected to NMR multi-echo image acquisition. Data analysis was performed utilizing MATLAB R2022a software.

Instrument model: The MRTrain05-90 MRI machine is a low-field magnetic resonance imaging device developed by the Institute of Electrical Engineering of the Chinese Academy of Sciences (IEE, CAS). It features a permanent magnet with 0.5 T magnetic field strength and a circularly polarized head-arrayed radiofrequency (RF) coil with a 4.5 cm aperture.

**Figure 2:**
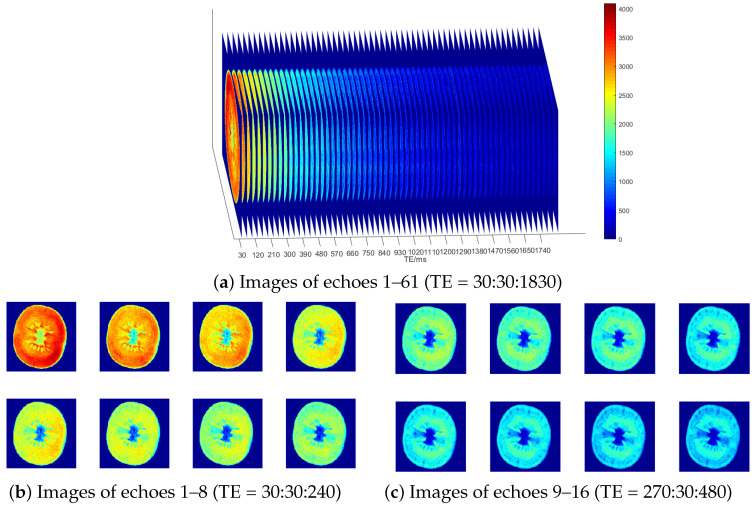
Multi-echo tomato image dataset.

Multi-echo Spin Echo (MESE) pulse sequences were utilized to non-destructively acquire multi-echo nuclear magnetic resonance images of the median planes (transverse sections) of tomato samples. The sequence parameters were set as follows: TR = 4000 ms (long TR time); ETL = 61; TE = 30, 60, 90, 120, …, 1830 ms; and slice thickness = 1.3 cm. Signal intensity (SI) was measured at different TE values for each voxel in the equatorial slice of the sample, generating a 61-echo dataset with a resolution of 256 × 256 pixels, as shown in Figure 2.

## 3. Data Processing

### 3.1. Nonlinear Diffusion Filter Denoising

**Figure 3:**
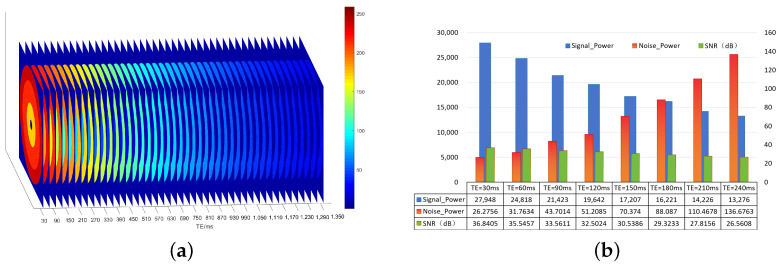
A diagram of simulated data and SNR analysis of each echo layer. (**a**) A diagram of simulated data (256 × 256 × 45). Inner loop: A = 250, T2 = 80 ms; Middle loop: A = 250, T2 = 200 ms; Outer loop: A = 250, T2 = 600 ms. (**b**) Analysis of signal and noise in each echo layer.

The main sources of noise in the process of MRI image acquisition include asymmetric magnetic fields, thermal noise, and vortices [35]. The main noise carried in the real and imaginary channels of the receiving coil collected by the device is generally considered to be independent additive Gaussian white noise with a mean value of 0 and a variance of $\sigma_{2}$ [33]. To obtain the final visual MRI image data, the real and imaginary data were reconstructed through modular operations, resulting in the transformation of the noise into a Ricean distribution [29]. This study constructed theoretical simulation data, quantitatively analyzed variations in the signal-to-noise ratio of each layer, and estimated and analyzed the signal and noise in multi-echo MRI image data in each layer, as shown in Figure 3. As TE increases, the signal power decreases, and the overall noise power increases; thus, the signal-to-noise ratio decreases layer by layer.

The denoising of multi-echo MRI images through filtering algorithms is crucial for subsequent structural segmentation, inversion operations, and quantitative analysis, as it maintains details while suppressing noise [33]. There is an exponential relationship between voxel points with the same two-dimensional coordinates in each layer of multi-echo MRI images (as shown in Equation (6)). The traditional method of layer-by-layer denoising is inadequate to utilize the strong correlation between layers in the image.

Based on non-local means (NLmeans) filtering (Equation (1)) and the strong layer-by-layer correlation of multi-echo images obtained from different research objects, as well as the increasing layer-by-layer heteroscedastic noise, a three-dimensional parameter te (representing different echo intervals) and increasing layer-by-layer local variance *h* are introduced (Equation (2)). A three-dimensional non-local mean denoising model based on NLmeans improvement (NLmeans3D) is adopted.

(1) $$v\left(i\right)=\sum_{j\in I}w\left(i,j\right)v\left(j\right),$$

(2) $$v\left(i,te\right)=\sum_{j\in I}w\left(i,j,te\right)v\left(j,te\right),$$

(3) $$w\left(i,j,te\right)=\frac{1}{z(i,te)}e^{-\frac{{∥\left({∥v\left(N_{i},te\right)-v\left(N_{j},te\right)∥}_{2,a_{1}}^{2}\right)∥}_{2,a_{2}}^{2}}{h^{2}}},$$

(4) $$z\left(i\right)=\sum_{j}w\left(i,j,te\right),$$

In Equation (3), the distance between v($N_{i}$,te) and v($N_{j}$,te) is a 3D voxel block. We calculate the structural differences between adjacent pixels in the te dimension using the classical 2-norm, enhance the temporal adjacency effect through the standard deviation $a_{1}$ Gaussian convolution, and then calculate the structural differences between positionally adjacent pixels using the classical 2-norm of v($N_{i}$) and v($N_{j}$). The positional adjacency effect is strengthened through the standard deviation $a_{2}$ Gaussian convolution. z(i,te) is a normalized parameter (Equation (4)), while *h* is a distortion measurement parameter between adjacent voxel strengths and gradually increases layer by layer based on the noise power acquired.

**Figure 4:**
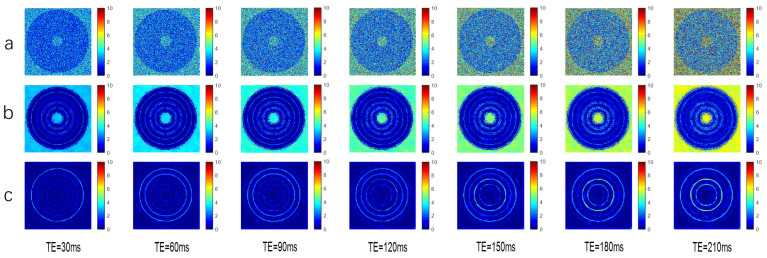
Diagram of simulated data and SNR analysis of each echo layer. (**a**) Noise graph, (**b**) Efficacy of layer-by-layer NLmeans filtering, (**c**) Efficacy of NLmeans_3D filtering.

The simulated data (Figure 4) demonstrate that the algorithm exhibits better objective denoising indicators and image detail processing capabilities than the layer-by-layer echo NLmeans denoising algorithm, meaning it can improve tissue characterization and increase the accuracy of the subsequent multi-exponential inversion algorithm.

### 3.2. Cherry Tomato Multi-Echo MRI Image Structure

#### 3.2.1. Analysis of Internal Structure of Cherry Tomatoes

**Figure 5:**
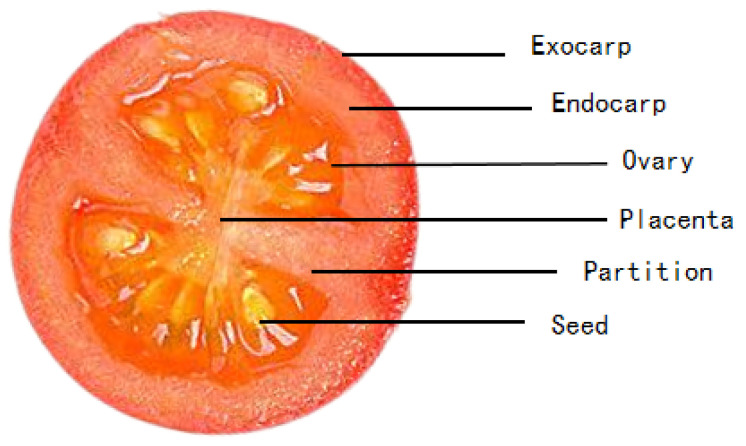
Tomato structure diagram.

The internal structure of the cherry tomato consists mainly of the pericarp, the internal tissue of the ovary, and the placenta. The pericarp consists of the exocarp, mesocarp, and endocarp. The endocarp and mesocarp are similar in structure and composition, with little apparent difference in appearance. The intra-ovary tissue is an important component of the pulp and contains the partitioning structures and gums that divide the chambers. The seeds inside the cherry tomato are usually located in the central part of the pulp and are surrounded by soft pectin. The placenta is the bottom part of the tomato fruit, also known as the fruit base or stalk area, as shown in Figure 5.

According to the internal structure of the cherry tomato, the endocarp and mesocarp were unified as the overall structure to facilitate the subsequent quantitative analysis. The exocarp, endocarp, partition structure, ovary tissue, seeds, and placenta were positioned sequentially from the inner to the outer edges, and the structural segmentation template of the multi-echo image of cherry tomato was established.

#### 3.2.2. Structural Segmentation Template Based on Deep Learning

The overlapping structures of tomato fruits and their blurred boundaries result in insufficient segmentation performance by traditional image segmentation algorithms. To achieve effective structural segmentation, retaining the segmented details and focusing on global features are indispensable to the model, which can fully and effectively segment the image structure while maximizing the preservation of the segmented image details.

The TransUNet [36] model, anchored by Transformer, makes the perception field self-adapting and performs favorably in difficult semantic tasks. However, Conv Block extracts basic information only, and the extraction of local information is inadequate. Therefore, this experiment focused on the diverse characteristics of multi-echo images with different structures, layers, and saliency. An improvement was introduced to perform the structural segmentation of cherry tomato multi-echo MRI images based on the TransUNet model.

**Figure 6:**
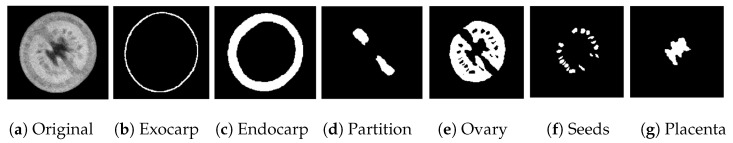
Segmentation results for each structure of cherry tomato.

On the basis of the TransUNet model, a local information supplementation module is attached to the Skip Connection using spatial and channel perception attention mechanisms. By introducing a hybrid module for convolution and SwinTransformer [16], utilizing the long-range perception ability of SwinBlock, and leveraging the advantages of the CNN, the semantic perception ability is improved, which maximizes the preservation of segmentation image details. The exocarp, endocarp, ovary, placenta, partition, and seeds are thus effectively segmented as cherry tomato structures (Figure 6).

### 3.3. Signal Modeling and Inversion Algorithms

#### 3.3.1. Physical Significance of T2 Relaxation

In nuclear magnetic resonance (NMR), T2 relaxation refers to the process by which protons, after being excited by a radiofrequency pulse, exchange energy due to interactions, leading to the decay of transverse magnetization to zero. The T2 relaxation times in fruits reflect the mobility and interaction strength of water molecules [37,38]. Different types of water within fruits exhibit varying mobility, resulting in distinct T2 relaxation times. Free water molecules, which are highly mobile, have larger T2 values, while bound water molecules, which are less mobile, have smaller T2 values. Some studies suggest that the magnitude of T2 represents the varying restrictions on water mobility and interactions imposed by the microenvironment and thus represents water located in different microenvironments, such as cell walls, the cytoplasm, and vacuoles. In previous research, T2 relaxation typically signifies water in different states (free water, semi-bound water, bound water) [39,40,41] or different subcellular compartments (vacuoles, cytoplasm, cell walls) [37,38] within the tissue.

#### 3.3.2. Signal Multi-Echo Attenuation Model

An NMR image is based on two physical processes, T1 relaxation and T2 relaxation, which occur simultaneously and independently of each other, and the signal is spatially encoded using a gradient field, setting TR (repetition time) and TE (echo time) to obtain the SIG (magnetized signal intensity) providing the sample’s spatial information on a voxel-by-voxel basis. The magnetized signal intensity (SI) represents the signal strength, and N(H) is the hydrogen proton density. In this experiment, TR = 4000 ms (much larger than T1), so the signal intensity at each voxel point is mainly determined by the hydrogen proton density and T2 of that voxel (Equation (5)).

(5) $$SIG\propto N\left(H\right)\left(e^{-\frac{TE}{T2}}\right)\left(1-e^{-\frac{TR}{T1}}\right)\propto N\left(H\right)\left(e^{-\frac{TE}{T2}}\right),$$

Without considering the effect of noise, the effective measured signal at moment t for each voxel *i* is represented by the T2 relaxation signal multi-exponential decay model.

(6) $$S_{it}\left(\theta ,t\right)=\sum_{1}^{k}A\left(e,j\right)e^{-\frac{t}{T_{2}\left(e,i\right)}},$$

In Equation (6), e represents the total number of exponential components. A(n,j) and T2(n,j) are the amplitude and relaxation time of each exponential component e in voxel j, and t is the echo time for the acquisition of the weighted image. In the present experimental environment, t(n) = 30 ms, 60 ms,…. The parameter θj = A(1,j), T2(1,j), …,A(e,j), T2(e,j) characterizes the T2 characteristics of voxel j of the sample. The mono-exponential decay model (e = 1) provides an overall relaxation time and corresponding amplitude for all components or water states within the tissue. In contrast, the multi-exponential decay model (e = 2, 3, 4) retrieves discrete parameters for various water state fractions, offering a richer representation of tissue information.

In this experiment, the echo data underwent a tri-exponential inversion, resulting in T2 (relaxation time) and A (water content) data. Based on the T2 relaxation time of water within the sample, water was categorized into three groups: freely mobile water (T23), less mobile water (T22), and bound water (T21). The corresponding amplitudes A03, A02, and A01 represent the relative proportions of water in each state.

T2 relaxation time varies along with the ripening of fruits, and the use of low-field NMR techniques to acquire images non-destructively for quantitative analysis can be used to obtain the cellular and tissue-level T2 relaxation times of samples from fruits in different stages of physiological changes or processing. Fruits can be continuously acquired to obtain sample cellular and tissue-level relaxation information and to study the changing laws of the structure and moisture components of fruit tissues in the development, ripening, and processing stages [17,42,43].

#### 3.3.3. T2 Inversion Evaluation Function

Solving the T2 relaxation signal in the presence of noise using multi-echo data can be reduced to a nonlinear multi-exponential signal inversion problem [38]. In this experiment, the classical evaluation function was used to minimize the squared difference between the fitted value of the multi-exponential model of the signal and the acquired (measured) value of the multi-echo signal using the least squares criterion. For the inversion object, the objective function is shown in Equation (7). The optimal T2 spectral inversion objective function transforms the solution of the system of equations [43] into an optimization problem. Minimizing the LS criterion (i.e., the squared difference) based on the multi-exponential fitting of the model between the signals and the measured data, the quadratic criterion minimization for each voxel is given by Equation (7), which corresponds to the measured values of the echo components of the attenuated signal in the containing voxel.

(7) $$C_{LS_{i}}\left(y_{i},\theta_{i}\right)=\frac{1}{2\sigma_{i}^{2}}\sum_{t=1}^{n}{\left[y_{it}-s_{it}\left(\theta_{i}\right)\right]}^{2},$$

#### 3.3.4. Chaotic Particle Swarm Inversion Algorithm Based on Adaptive Component Segmentation

Traditional optimization techniques for addressing the aforementioned least squares problem encompass Newton’s method, Singular Value Decomposition, and the Levenberg–Marquardt algorithm. A common characteristic of these methods is their sensitivity to parameter configurations; varying initial values and iteration step sizes can lead to divergent solutions, potentially resulting in local optima [43].

To address the challenge of variable optimal initial values and component intervals for samples across various maturation stages and structural inversion processes, the Chaotic Immune Particle Swarm Optimization (ACS-CIPSO) algorithm has been refined with Adaptive Component Segmentation interval-setting limits. This enhancement integrates cloning, crossover, mutation, and receptor correction strategies from the Artificial Immune System into the particle swarm optimization framework through the CIPSO algorithm [44]. Furthermore, a chaos operator is leveraged to perform mutation and to make multidimensional adaptive adjustments in the inertia factor, as well as the upper and lower bounds of the multi-exponential intervals.

The particle swarm optimization algorithm (PSO) treats each feasible solution as a particle with two parameters: position and velocity. During the iteration process, the fitness function of each particle is calculated, and then the particle swarm constantly tracks the best particles. The velocity *v* and position of a particular particle are updated according to the following Equation (8):

(8) $$v=wv+c_{1}r_{1}\left(pBest-x\right)+c_{2}r_{2}\left(nBest-x\right),$$

pBest is the best position experienced by the current particle, and nBest is the best particle in the neighborhood of the current particle. If the neighborhood is the entire particle population, nBest is the global best particle, called gBest. w is the inertia weight, which controls the effect of the past velocity on the present velocity.

The algorithm introduces an Artificial Immune System (AIS), which allows a particle to be cloned according to its affinity, and a chaos operator to generate new particles, which introduces crossover and mutation factors to ensure the traversal of the particles in the solution space as well as particle independence.

(9) $$\left\{\begin{array}{c} r_{1}\left(t+1\right)=4r_{1}\left(t\right)\left[1-r_{1}\left(t\right)\right]\\ r_{2}\left(t+1\right)=4r_{2}\left(t\right)\left[1-r_{2}\left(t\right)\right] \end{array}\right.,$$

The improved algorithm introduces adaptive interval segmentation, which sets the inertia factor for multidimensional adaptive adjustment of the interval traversal range (Equation (9)) to overcome the floating of component interval ranges on the inversion results.

(10) $$\left\{\begin{array}{c} \theta_{\max}={\left[a_{11},t_{11},a_{12},t_{12},a_{13},t_{13}\right]}_{\max}\\ \theta_{\min}={\left[a_{21},t_{21},a_{22},t_{22},a_{23},t_{23}\right]}_{\min} \end{array}\right.,$$

The algorithm introduces adaptive interval segmentation. Taking the tri-exponential signal model as an example, the upper limit and lower limit of the interval range (Equation (10)) include three non-overlapping relaxation intervals and corresponding amplitudes. The component interval ranges are based on the group’s physical meaning and individual characteristics of the sample objects, and an unreasonable interval range setting will fall into a local optimal solution. The peak correction strategy is used to set the upper limit (Equation (11)) and lower limit (Equation (12)) of the compression or amplification interval range using the compression and amplification factor.

(11) $$\left\{\begin{array}{c} m_{1}=\theta_{\max}\left(n\right)-\theta \left(n\right)\\ \theta_{\max}\left(n+1\right)=\theta_{\max}\left(n\right)-\ln\left(\frac{m_{1}}{\gamma }\right)m_{1}>s,m_{1}<1 \end{array}\right.,$$

(12) $$\left\{\begin{array}{c} m_{2}=\theta \left(n\right)-\theta_{\min}\left(n\right)\\ \theta_{\min}\left(n+1\right)=\theta_{\min}\left(n\right)-\ln\left(\frac{m_{2}}{\gamma }\right)m_{2}>s,m_{2}<1 \end{array}\right.,$$

$\theta_{max}$ and $\theta_{min}$ denote the arrays of maximum and minimum values stored in the tri-exponential inversion with respect to each of the six preset parameter values, and *s* is the threshold for interval updating.

**Figure 7:**
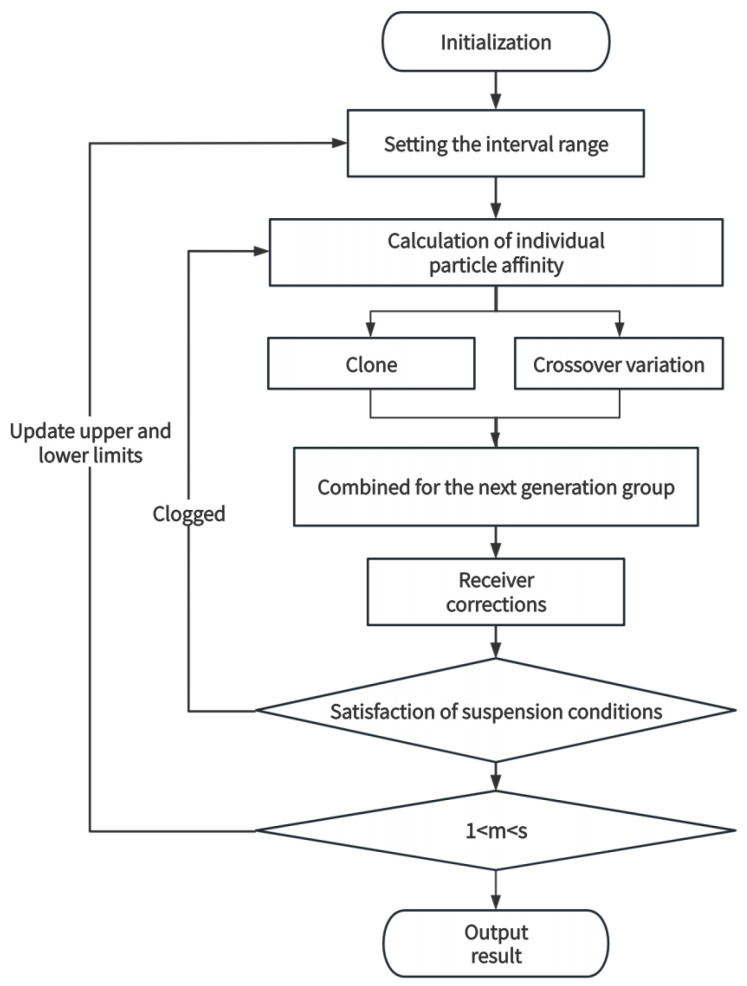
ACS-CIPSO inversion algorithm program flow chart.

The flow chart of the procedure (Figure 7), which introduces the crossover and variation factors, ensures the traversal of particles in the solution space as well as inter-particle irrelevance, while the multidimensional adaptive adjustment of traversal intervals overcomes the impact of the fluctuation of the range of intervals of individual sample components on the inversion results.

## 4. Results

### 4.1. T2 Inversion of Multi-Structure Echo Data

Using the six-structure template established in Figure 6, the mean value of the 64-echo signal was extracted from each sample for the six structures. Based on the signal multi-echo decay model (Equation (7)), echo exclusion processing was employed to process echoes and exclude anomalies caused by interference and noise. The T2 relaxation time of each structure and its corresponding amplitude (A) were inverted according to the signal multi-echo decay model, and the ACS-CIPSO inversion algorithm (Figure 7) was used to perform mono-exponential and tri-exponential inversion of the structural data for each sample. The inversion error data were analyzed, as shown in Table 1.

### 4.2. Analysis of Results of Mono-Exponential Inversion

After inverting the mono-exponential T2 of structured multi-echo data from multiple samples across three varieties and four stages of ripening, the overall pattern of T2 value changes associated with the maturation process in six distinct fruit structures was investigated. In mono-exponential inversion, the T2 inversion results are considered as a whole, and the output results are single T2 values, which can reflect the overall change trend of the internal structural moisture of tomato fruits as a whole. The inversion outcomes for different varieties and maturities, along with their corresponding T2 and A, are depicted in six charts representing the green ripening, color change, firm ripening, and full ripening stages for varieties A, B, C, and D.

**Figure 8:**
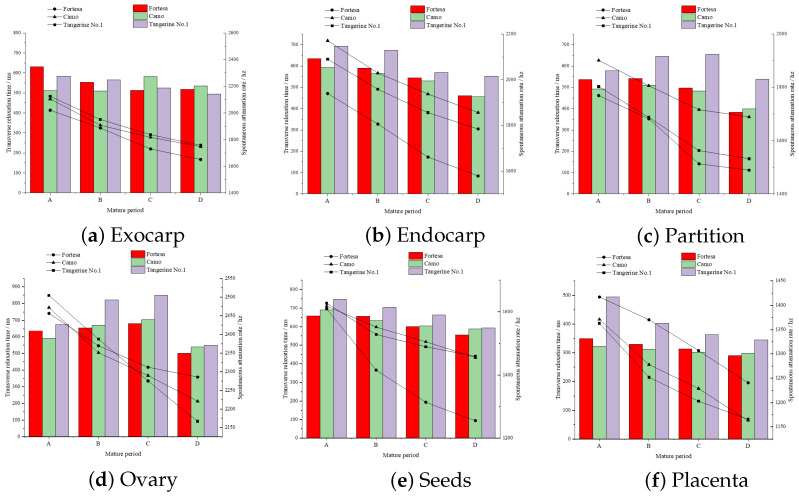
Mono-exponential T2 inversion results for six structures. The line charts represent A. The bar charts represent T2.

The overall water mobility in all tomato fruit samples showed a significant decrease in the “full ripening” stage (confirming the hypothesis that T2 decreases in the full ripening stage [3]), with differential changes observed during the intermediate stages. Notably, within the endocarp, seeds, and placenta structures, the degree of mobility continued to decline, with the endocarp (Figure 8b) showing a more substantial decrease compared to the seeds (Figure 8e) and placenta (Figure 8f), with an average reduction of 23.6%. Variations in the exocarp were observed among different varieties. The degree of mobility in the partition (Figure 8c) and ovary (Figure 8d) structures initially increased slightly and then decreased in the “full ripening” stage, exhibiting a fluctuating pattern. Water content showed a continuous decrease in the six middle structures, with larger decreases of 22.1% in the seeds and 18.6% in the endocarp. All value comparisons are made relative to the initial detection stage, known as green ripening (A stage).

**Figure 9:**
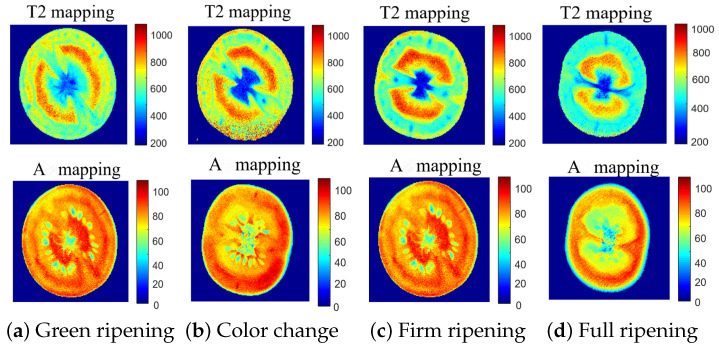
T2 and A mapping of Tangerine No. 1 cherry tomatoes at four ripening stages.

Utilizing Equations (9) and (10), voxel-wise mono-exponential inversion was conducted on a series of cherry tomato samples (Tangerine No. 1), resulting in voxel-specific relaxation times (T2 mapping) and amplitudes (A mapping) for the four distinct ripening stages, as depicted in Figure 9. This approach facilitated a direct comprehension of the alterations in water mobility (T2 mapping) and content (A mapping). Furthermore, the analysis distinctly revealed the influence of noise on the data within voxel-wise processing (Figure 9b). A comparative analysis of the example samples with structural changes showed that trends in water mobility and water content corresponded with the structural changes.

As shown in Figure 9, the T2 mapping of the samples showed a noticeable decrease in T2 values in the full ripening period, with a continuous reduction in water content across the four stages. This consistency validates the accuracy and effectiveness of the structured analysis method and provides compelling evidence for understanding the dynamics of water during the ripening process of cherry tomatoes.

The single-component T2 can only present the overall trend in water changes and cannot visually depict the multi-component information on water in tomato fruit, which is a microstructure.

### 4.3. Analysis of Results of Tri-Exponential Inversion

**Figure 10:**
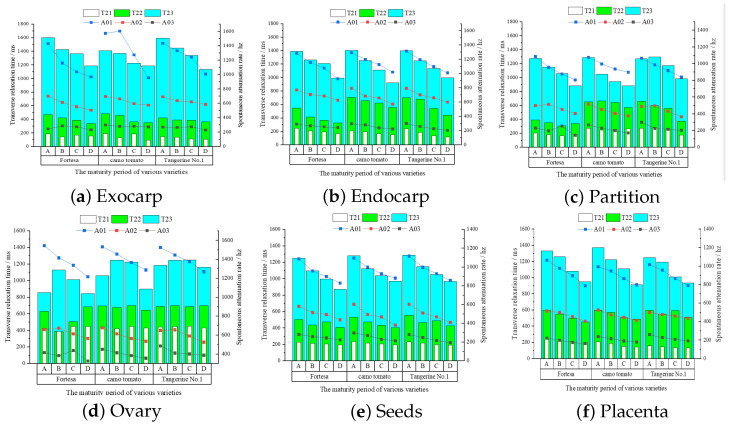
Tri-exponential T2 inversion results for six structures.

The tri-exponential T2 inversion of structured multi-echo data from multiple samples was conducted to discern the changing patterns of three distinct water states and their contents within the six structures throughout the ripening process. The inversion results of the six structures of the three varieties and four ripening stages are shown in Figure 10. A, B, C, and D represent the four ripening stages, namely, green ripening, color change, firm ripening, and full ripening, respectively. The T2 values are represented by three components: T21, T22, and T23, which denote bound water, semi-bound water, and free water, respectively. A01, A02, and A03 indicate the respective proportions, representing the contents of the corresponding water states [16,33].

Although all three types of water with differing mobility continued to decrease in both the endocarp (Figure 10b) and exocarp (Figure 10a) structures, the greatest decrease in bound water was found in the inner and outer pericarp at 34.3% and 28.8%, and the water content showed a tendency to decrease, with the most significant decreasing tendency observed for free water at 31.8% and 32.3%, respectively. In contrast, fluctuations in the exocarp water content may be attributed to its small area, which makes it prone to segmentation errors.

The degree of water mobility within the partition (Figure 10c) and ovary (Figure 10d) fluctuates downward, and the water content variation within the locules is complex. From stage A to stage D, there is a consistent decrease in the free water content (A03) in both the partition and ovary, with reductions of 21.2% and 16.8%, respectively. Meanwhile, the levels of bound (T21) and semi-bound (T22) water exhibit fluctuations.

The three phases of water mobility in the seed (Figure 10e) and placenta (Figure 10f) structures tended to decrease during growth, with the most significant decreases in bound-water mobility being 33.5% and 30.4%, and the contents of the three water types continued to decrease, with the decrease in free water content being the most significant at 23.8% and 26.1%.

## 5. Discussion

This study aimed to deeply explore the changes in moisture mobility and its migration throughout the ripening process of cherry tomatoes by utilizing qMRI technology. Addressing the challenge of low signal-to-noise ratios in multi-echo MRI images from low-field equipment, this study introduces an innovative solution.

In this study, a specialized 3D non-local means filtering algorithm (NLmeans3D) was developed for multi-echo data. Concurrently, the TransUNet model was enhanced to successfully segment six key structures within cherry tomato MRI images, leading to the creation of six structural templates for extracting the corresponding T2 echo data from each structure. Subsequently, the ACS-CIPSO algorithm was employed to conduct both mono-exponential and tri-exponential inversions of the echo data, thereby enhancing the accuracy of the analysis. The structured T2 relaxation detection method effectively mitigates the noise errors from voxel-by-voxel processing and maintains the relaxation variability across structures. By analyzing three varieties and four ripening stages, this study quantitatively revealed the moisture component changes in cherry tomatoes, providing crucial data and a theoretical foundation for understanding moisture dynamics in ripening fruits.

The results of the mono-exponential analysis indicate a significant decrease in water mobility (T2) in the final detection stage (full ripening, stage D) of tomato fruit, with differential changes observed in intermediate stages. Particularly in the endocarp, seed, and placenta structures, there is a continuous decline in mobility, with the endocarp showing the most significant reduction, with an average decrease of 23.6%. Variations exist among different varieties in the exocarp, while the mobility in the ovular and septal structures exhibits fluctuating behavior. Water content (A) persistently decreases across all six structures, with particularly notable reductions in the seed and endocarp structures. The tri-exponential analysis further clarifies the patterns of changes in water with different degrees of mobility and content. Across the four ripening stages, there is a continuous and significant decline in the mobility of free water (T23) and bound water (T21) in the endocarp, seed, and placenta structures. These findings provide profound insights into the changes in water mobility during the ripening process of tomato fruit.

## 6. Conclusions

This study employed quantitative magnetic resonance imaging (qMRI) technology to conduct a quantitative analysis of the moisture mobility and content within different structures of cherry tomatoes throughout their ripening process. The results indicate that during ripening, there is a regular trend in the mobility and content of moisture within various structures of cherry tomatoes. Additionally, there are regular differences across different structures during the ripening process.

The findings of this study reveal the patterns of moisture redistribution in cherry tomatoes during ripening, offering a novel approach to the precise determination of fruit ripeness. By jointly analyzing the mobility and content of free and bound water in the pericarp, seeds, and placenta, the ripeness of cherry tomatoes can be effectively assessed. This provides a basis for the quality assessment of fruits and opens a new and effective research pathway for the determination of their ripeness.

This study’s methods and findings contribute to gaining a deeper understanding of fruit respiration types, ripening regulation, and the formation and maintenance of storage quality. For instance, the patterns of moisture migration discovered in this study can provide clues about the relationship between moisture and respiratory metabolism during fruit ripening, thereby offering a theoretical basis for optimizing fruit storage and preservation techniques.

Future research can enrich the types of data and explore the moisture migration patterns of cherry tomatoes under different environmental conditions, across various cultivars, and at multiple ripening stages, aiming to establish more accurate ripeness assessment models. Moreover, the methods used in this study can be integrated with other non-destructive testing technologies, such as spectral analysis and acoustic detection, to construct a multi-parameter integrated quality detection platform for more comprehensive and precise quality assessment.

The outcomes of this study not only provide a new technical means for determining the ripeness of cherry tomatoes but also offer significant theoretical foundations and data support for the research on fruit quality assessment and preservation techniques, playing a crucial role in advancing the development of the fruit industry.

## Figures and Tables

**Table 1:** Mono-exponential and tri-exponential T2 inversion error analysis of each structure. Raw: Result before exclusion processing; Exp: result after exclusion processing; R2: Coefficient of Determination; RMSE: Root Mean Square Error.

Structure	R2 (Raw/Exp) Mono-Exp	RMSE (Raw/Exp) Mono-Exp	R2 (Raw/Exp) Tri-Exp	RMSE (Raw/Exp) Tri-Exp
Exocarp	0.985/0.995	0.103/0.080	0.985/0.994	0.116/0.075
Endocarp	0.988/0.998	0.115/0.045	0.986/0.997	0.101/0.049
Partition	0.983/0.994	0.102/0.083	0.983/0.993	0.103/0.082
Ovary tissue	0.984/0.990	0.105/0.087	0.983/0.992	0.102/0.085
Seeds	0.988/0.998	0.098/0.048	0.987/0.996	0.099/0.058
Placenta	0.987/0.996	0.098/0.052	0.986/0.997	0.102/0.051

## Data Availability

The original contributions presented in the study are included in the article, further inquiries can be directed to the corresponding authors.

## Abbreviations

The following abbreviations are used in this manuscript:

qMRI	Quantitative magnetic resonance imaging
T2	T2 relaxation inversion
TR	Repetition time
TE	Echo time
SI	Magnetized signal intensity
ETL	Echo Train Length
NLmeans	Non-local means
LD	Linear Dichroism
